# Incorporating a Novel Dual Transfer Learning Approach for Medical Images

**DOI:** 10.3390/s23020570

**Published:** 2023-01-04

**Authors:** Abdulrahman Abbas Mukhlif, Belal Al-Khateeb, Mazin Abed Mohammed

**Affiliations:** Computer Science Department, College of Computer Science and Information Technology, University of Anbar, Ramadi 31001, Anbar, Iraq

**Keywords:** transfer learning, fine-tuning, data augmentation, skin cancer, breast cancer, imbalanced datasets, medical images

## Abstract

Recently, transfer learning approaches appeared to reduce the need for many classified medical images. However, these approaches still contain some limitations due to the mismatch of the domain between the source domain and the target domain. Therefore, this study aims to propose a novel approach, called Dual Transfer Learning (DTL), based on the convergence of patterns between the source and target domains. The proposed approach is applied to four pre-trained models (VGG16, Xception, ResNet50, MobileNetV2) using two datasets: ISIC2020 skin cancer images and ICIAR2018 breast cancer images, by fine-tuning the last layers on a sufficient number of unclassified images of the same disease and on a small number of classified images of the target task, in addition to using data augmentation techniques to balance classes and to increase the number of samples. According to the obtained results, it has been experimentally proven that the proposed approach has improved the performance of all models, where without data augmentation, the performance of the VGG16 model, Xception model, ResNet50 model, and MobileNetV2 model are improved by 0.28%, 10.96%, 15.73%, and 10.4%, respectively, while, with data augmentation, the VGG16 model, Xception model, ResNet50 model, and MobileNetV2 model are improved by 19.66%, 34.76%, 31.76%, and 33.03%, respectively. The Xception model obtained the highest performance compared to the rest of the models when classifying skin cancer images in the ISIC2020 dataset, as it obtained 96.83%, 96.919%, 96.826%, 96.825%, 99.07%, and 94.58% for accuracy, precision, recall, F1-score, sensitivity, and specificity respectively. To classify the images of the ICIAR 2018 dataset for breast cancer, the Xception model obtained 99%, 99.003%, 98.995%, 99%, 98.55%, and 99.14% for accuracy, precision, recall, F1-score, sensitivity, and specificity, respectively. Through these results, the proposed approach improved the models’ performance when fine-tuning was performed on unclassified images of the same disease.

## 1. Introduction

**Figure 1:**
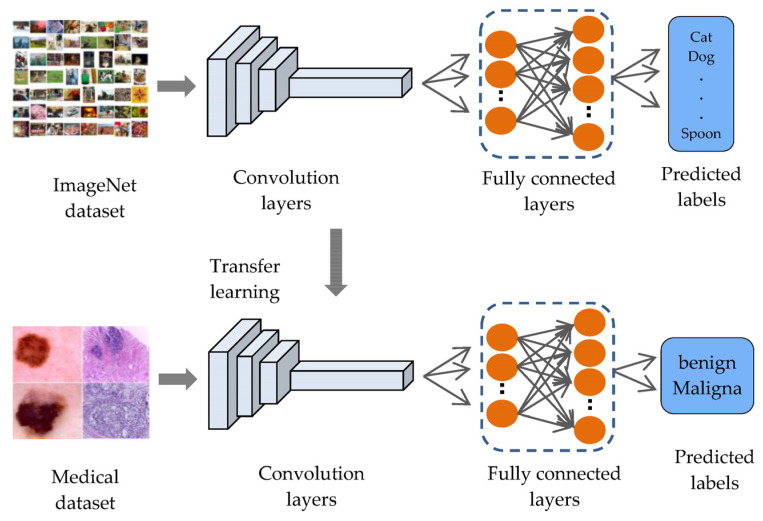
Transfer learning from ImageNet.

Although there are many machine learning techniques to analyze medical images in various areas, deep learning has become the better method to analyze and interpret medical issues due to its accuracy [1]. Deep learning is a part of machine learning and is based on artificial neural networks, called deep neural networks because the structure of the neural network consists of multiple inputs, outputs, and hidden layers [2]. Deep learning is widely known for its application in many areas, and is most important in the analysis and interpretation of medical images [3], such as classifying melanomas [4,5], brain tumors [6,7], and eye diseases [8,9], to overcome image processing barriers and machine learning methods, although these applications also produce low-level classification accuracy with deep learning due to deep learning models needing a sufficient number of labeled images to perform better [10]. This will lead to a problem in the performance of deep learning in some fields, especially in the medical field, where the field of medical image analysis suffers from a lack of labeled images, due to the time-consuming and expensive process of labeling images, which requires experts specialized in radiology [10]. These reasons lead researchers to build computer systems that help experts make decisions and speed up the diagnostic process. Transfer learning is provided to reduce the need for many images and to speed up the training process by transferring knowledge from a previous process and then training it to relatively small datasets for the current task. Transfer learning is often applied to pre-trained models (such as LeNet, Alex-Net, VGG-16, ResNet, etc.) on the ImageNet dataset, which consists of natural images, with large numbers of more than 14 million images distributed over 1000 classes [11], such as objects, animals, and humans, to solve. Many tasks are pattern recognition and computer vision. For example, applying transfer learning on ImageNet (face detection, distinguishing types of animals, or distinguishing types of flowers, etc.) can improve the performance of these tasks, because their features are like those in the ImageNet dataset. However, the ImageNet dataset does not contain medical images, resulting in a domain mismatch between the source domain and the target domain as shown in Figure 1.

In addition, fine-tuning the models for field convergence requires more images due to increasing the number of trainable layers [12], which causes the problem of overfitting that occurs when models are trained on few images [13]. Moreover, medical datasets have a shortage of images in the malignant class compared to the number of images in the benign class, which causes an imbalance between the classes of the dataset [14], and thus causes the model bias problem for the class with the largest number of images. 

Deep learning has become the ultimate method for the examination and classification of cancerous diseases, due to its exactness, as there have been many previous works on deep learning approaches, especially transfer learning technology from the pre-trained models such as LeNet, Alex-Net, VGG-16, ResNet, etc. All related works are summarized in Table 1. V. Shah et al., 2020 [15], used the models (DenseNet-121, SE-ResNeXt50, ResNet50, and VGG19) to classify the ISIC2020 melanoma dataset images into malignant and benign. ResNet-50, according to sensitivity, specificity, and accuracy, obtained the best results among the other three, with values of 99.7%, 55.67%, and 93.96%, respectively. It is pointless to use a test with low specificity for diagnosis because many people without the disease will show positive results and potentially receive unnecessary diagnostic procedures. C. Li et al., 2021 [16], applied transfer learning on three models (EfficientNet-B4, vgg16, and ResNet50) for the purpose of classifying melanoma images in the ISIC2020 dataset. They use data augmentation to improve the performance and accuracy of the model; after the training procedure, they had an AUC-ROC score for EfficientNet-B4 of 0.909, which is 3.5% higher than VGG16 and 2.3% higher than Resnet50. They did not experiment with the effect of balancing the classes, because the ISIC2020 dataset suffers from the problem of imbalance between the benign and malignant classes. In addition, the proposed model suffers from the problem of overfitting. R. Zhang, 2021 [17], used the EfficientNet-B6 model and performed a transfer learning of the model on the ISIC2020 dataset. He obtained an AUC-ROC score of 0.917. His model suffered from an overfitting problem. Z. M. Arkah et al., 2021 [18], proposed a new approach to transfer learning by training the models (VGG, GoogleNet, ResNet50) from scratch on a large number of unlabeled melanoma images, and then training them on a small number of labeled skin images. They applied their approach to the ISIC 2020 dataset. The ResNet50 achieved an accuracy of 93.7% when training with the proposed method. However, training the models from scratch takes time and requires a very large number of images, so the process of fine-tuning the pre-trained models to some last layer that extracts custom features may lead to better results and less training time. 

L. Alzubaidi et al., 2021 [10], proposed a new model that combines recent advances, trained it from scratch on large datasets of unlabeled medical images, and retrained the model classifier on a small number of labeled images. They applied the model to the ISIC2020 dataset, in addition to using data augmentation techniques, to increase the number of samples. They have experimentally demonstrated that the proposed method can significantly improve the classification performance. The proposed model achieved an F1 score of 98.53% with the proposed method. The process of training from scratch performs better, but it takes a lot of time to train, requires a lot of images to practice well, and you may run into the problem of overfitting that often occurs when designing new models. R. Kaur et al., 2022 [19], proposed a DCNN that is lightweight and less complex than other recent approaches to classify melanomas with high efficiency. In their study, the model was tested on various cancer samples from the International Skin Imaging Collaboration data stores (ISIC 2016, ISIC2017, and ISIC 2020). The proposed DCNN achieved an average accuracy of 81.41% on ISIC 2016, an 88.23% on ISIC 2017, and 90.48% on ISIC 2020. The designed DCNN model can be further extended to multi-class classification to predict other different types of skin cancers.

S. H. Kassani et al., 2019 [20], proposed a transfer-learning method on the Xception model to classify the ‘hematoxylin’ and ‘Eosin’ (H&E) spots available for histological breast cancer images in the ICIAR 2018 dataset. To improve performance, they used different stain normalization methods (Reinhard and Macenko). Various data augmentation methods were applied to increase the number of samples. Their proposed model had an average accuracy of 92.50%. The accuracy measure alone is not sufficient to evaluate the model, so other measures such as precision, recall, and F1 score can be used. T. Kausar et al., 2019 [21], used the VGG16 model to extract features and classify the histological images of breast cancer in the ICIAR2018 dataset. They have normalized H&E images by the Macenko method, as well as by using various methods of data augmentation techniques. Their model is based on images, as opposed to the models that based on patches, so they extracted features from 2048 × 1536 full size images. After that, a SoftMax classifier was trained on the extracted feature set. During their experiments, they achieved an accuracy of 94.3% for multi-category classification. The effect of data set size with or without data augmentation on classification has not been reported. C. P. Nguyen et al., 2019 [22], solved the problem of the limited number of images in the ICIAR2018 target dataset. To improve classification accuracy, they performed augmentation of the data in the test phase. They obtained a result with 78% accuracy in predicting the test set from four classes. Data augmentation techniques using GAN to generate additional datasets have not been considered. L. Alzubaidi et al., 2021 [10], sliced all breast cancer histological images in the ICIAR-2018 dataset into 12 non-overlapping patches of 512 × 512 pixels to increase the number of images. Their method achieved an accuracy value of 97.51%. Despite the good results, the process of slicing the image into patches can miss some important information needed to correctly predict the category.

To avoid slicing the images into small patches that may lose some important information to the histological images, data augmentation techniques have been applied only to the entire image, to increase the number of samples and to extract sufficient patterns from the image.

**Figure 2:**
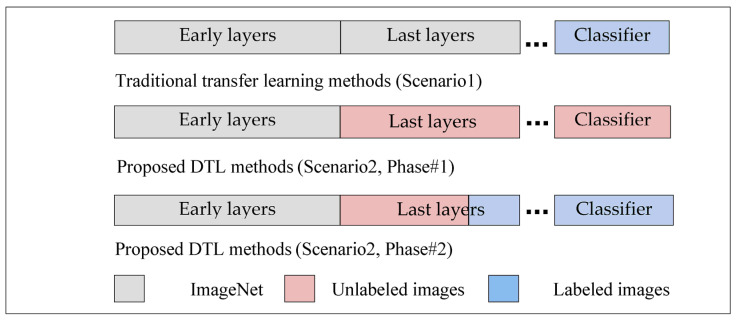
The difference between traditional transfer learning and proposed DTL methods.

Based on previous studies, it has been noted that all traditional transfer learning methods depend on pre-trained models on the ImageNet, which were used to extract features from them and to take advantage of the knowledge gained from them to classify the images of the new task, and this is not considered logical, because the ImageNet dataset includes natural images, and not medical images, to extract important features that can be used to support the task of classifying targeted medical images. Except for L. Alzubaidi et al., 2021, they trained a model from scratch on unclassified medical images of the same disease and applied transfer learning to classified images, but the training process from scratch also requires the presence of many images in addition to it taking time to train the model. To the best of our knowledge, this is the first work that aims to converge the domains between the source domain and the target domain by unfreezing the last layers that specialize in extracting special features, training them on unclassified medical images of the same disease, and training the classification layers on classified images of the target task, as shown in Figure 2. This process does not require many images and it does not require training the model from scratch. In addition, most of the previous studies were suffering from the problem of overfitting, so dropout layers by 50% are added to reduce this problem, in addition to using data augmentation techniques to increase the number of samples.

This study aims to converge the domain between the source domain and the target domain by taking advantage of the presence of large quantities of unclassified images of the same type of disease of the target task, and by proposing a novel methodology for transfer learning by fine-tuning the last layers on a large number of unclassified images of the same disease, and on a small number of classified images for the target task, in addition to solving the problem of unbalanced classes. Below is a summary of the most important contributions of this study:

1. Four novel models were designed based on pre-trained models (Vgg16, Xception, ResNet50, and MobileNetV2), and new layers were added to improve the prediction and classification process, as well as to solve the problem of overfitting.
2. Proposing a novel approach to transfer learning called DTL to solve the issue of the inefficiency of classified medical images, and the convergence of the field between the source domain and the target domain, by fine-tuning the last layers of the models on unclassified medical images of the same disease and then conducting the transfer learning again on a few classified images, which reduces the need for a large number of classified images. In addition to addressing the problem of the field convergence, because the features extracted from ImageNet are different from the features extracted from the target images.
3. Using a new method for pre-processing classified breast cancer images, by inserting the entire image into the model, without cropping the images into small patches (patch-wise), in order to preserve important patterns that may be lost while cropping the image into small patches.
4. Various data augmentation techniques to overcome the problem of unbalanced data and to increase the number of samples is applied.
5. To demonstrate that transfer learning from the same domain of the target dataset can significantly improve performance.
6. To validate the validity of the proposed models, they were tested on different medical imaging applications (skin cancer images and breast cancer images) as an example for the purpose of generalization.

The rest of the paper is organized as follows: Section 2 explains the materials and methods. Section 3 reports the results and discussions. Lastly, Section 4 concludes the paper.

## 2. Materials and Methods

**Figure 3:**
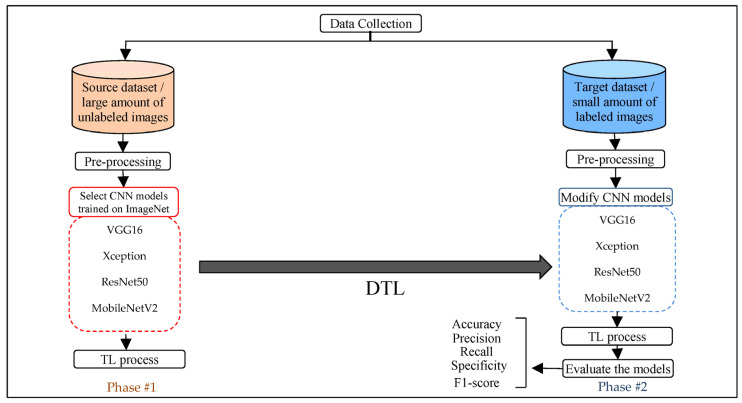
Steps and general framework for the proposed approach.

The transfer learning approach is one of the most important approaches to solving the problem of the lack of training data. However, there are still some limitations because the features gained from pre-trained models are considered generic and not close to the target task. Therefore, the last layers of the models are unfreezing to fine-tune the models on unlabeled images (to extract relevant features closest to the target task) and on labeled images for a few parts of the last layers. Moreover, modifying the fully connected layers increases the efficiency of the model’s performance, in addition to applying the data augmentation process for the purpose of balancing the images between the classes and increasing the number of samples for the purpose of training. This section presents all the steps and procedures, as shown in Figure 3, to achieve the proposed approach.

### 2.1. Data Collection

Several publicly available datasets for both diseases, skin cancer and breast cancer, are collected in the form of two sets: source dataset and target dataset. All the used datasets are described below.

- Source dataset

Source dataset includes unlabeled images; this set represents the source domain. For skin cancer, the first dataset is ISIC2019 that contains 33,569 images of dermoscopy images [23] with different image sizes: 1024 × 1024, 1024 × 680, 1024 × 681, 1024 × 682, 1024 × 674, 1024 × 764, 1024 × 768, 600 × 450, and 919 × 802, in jpg format. The second dataset is ISIC2020, which consists of 33,126 samples of dermatoscopy gathered from over 2000 patients [23], with large image sizes of 6000 × 4000, 4288 × 2848, and 3264 × 2448, in addition to different image sizes of 1920 × 1080 and 640 × 480, in jpg format. The third dataset is Derm7pt [24] Seven-Point Checklist Dermatology source dataset, abbreviated as derm7pt. This dataset contains about 2000 dermatoscopy images, with an image size of 768 × 512 in jpg format. The fourth dataset is PH2 [25], which consists of 200 dermoscopic images of melanocytic lesions. The dermoscopic images were obtained at the Dermatology Service of Hospital Pedro Hispano (Matosinhos, Portugal), under the same conditions, through the Tuebinger Mole Analyzer system using a magnification of 20×. They are 8-bit RGB color images with a resolution of 768 × 560 pixels in the file format BMP. The fifth dataset is PAD-UFES-20 [26], which contains 2298 samples. This dataset includes different resolutions, sizes, and lighting conditions. All images are available in PNG file format. The sixth dataset is MED-NODE [27], which contains 170 dermoscopic images from the digital image archive of the Department of Dermatology of the University Medical Center Groningen. This dataset includes different image sizes of 2000 × 1402, 2000 × 1583, 2000 × 907, 1199 × 907, 1200 × 1360, 841 × 759, and 781 × 704, in jpg file format. To become the total images in source dataset is 71,300 unlabeled images for skin cancer. 

For breast cancer, the first dataset is BreakHis [28], which contains 7909 images of breast cancer collected from 82 patients and magnified 40×, 100×, 200×, and 400×. All images are available with the size of 700 × 460 pixels and file format PNG. The second dataset is IDC [29]; this dataset includes histopathology micrographs from 922 images relating to 124 patients with IDC. This dataset includes different image sizes of 4032 × 3024, 2100 × 1574, and 1276 × 956, in jpg file format. The third dataset is SPIE-AAPM-NCI BreastPathQ [30]. This consists of 3698 image patches selected from whole slide images acquired from 64 patients from Sunnybrook Health Sciences Centre, with funding from the Canadian Cancer Society, and was made available for the BreastPathQ challenge sponsored by the SPIE, NCI/NIH, AAPM, and the Sunnybrook Research Institute. These image patches were a size of 512 × 512, which was then saved as uncompressed TIF image files. The fourth dataset is BreCaHAD [31]. This dataset contains 162 images of breast cancer histopathology images that each measure 1360 × 1024 pixels, and all images are available in TIF file format. The total images in source dataset is 12,691 unlabeled images for breast cancer images. For fine-tuning the model on the source dataset, each dataset is taken as a class within the source dataset and with the same name as the original dataset, because the purpose here is to train the model on images from the domain and not for classification.

- Target dataset

**Figure 4:**
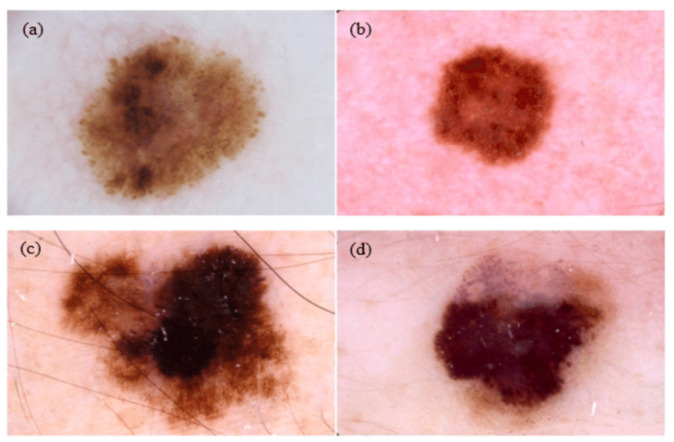
Examples of dermoscopy images: (**a**,**b**) are benign lesions; (**c**,**b**) are malignant lesions [33].

**Figure 5:**
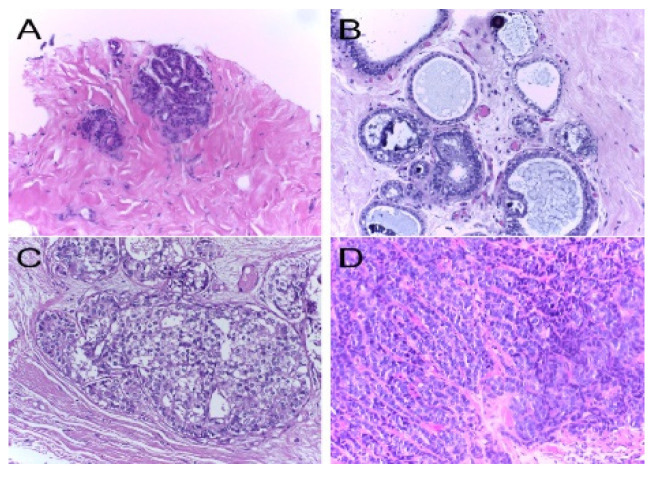
Examples of H&E stained images from the ICIAR2018: (**A**) normal tissue; (**B**) benign lesion; (**C**) in situ carcinoma; (**D**) invasive carcinoma. Hematoxylin stains the nuclei purple while eosin stains the stroma pink [34].

The target dataset includes labeled images; this set represents the target domain. For skin cancer, target dataset contains the SIIM-ISIC2020 [23] dataset that is classified into two classes: benign, which contains 32,542 images, and malignant, which contains 584 images (See Figure 4). For breast cancer, the target dataset contains ICIAR 2018 [32], which is composed of microscopy images of breast cancer annotated image-wise by two expert pathologists from the Institute of Molecular Pathology and Immunology of the University of Porto (IPATIMUP) and from the Institute for Research and Innovation in Health (i3S). This dataset includes 400 images with the size of 2048 × 1536 pixels. All images are available in TIF file format (See Figure 5).

### 2.2. Pre-Processing

**Figure 6:**
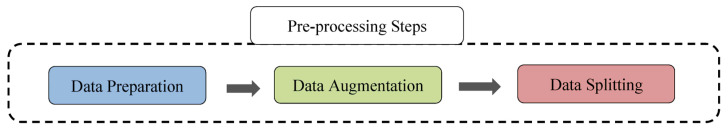
The pre-processing steps.

This section explains the operations that are performed on the datasets that are used in the proposed work, such as data preparation, data augmentation, and data splitting, for the purpose of initializing them before passing them to the model for the purpose of the training process. The pre-processing steps are described in Figure 6.

#### 2.2.1. Data Preparation

**Figure 7:**
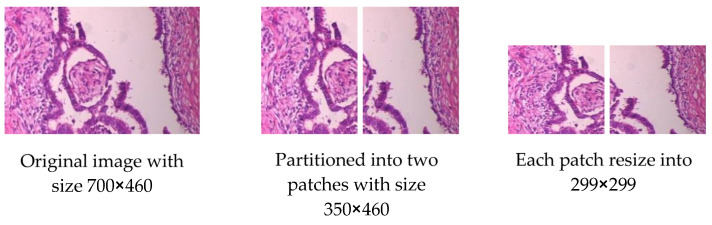
Example for Partitioning and resizing a BreakHis dataset.

**Figure 8:**
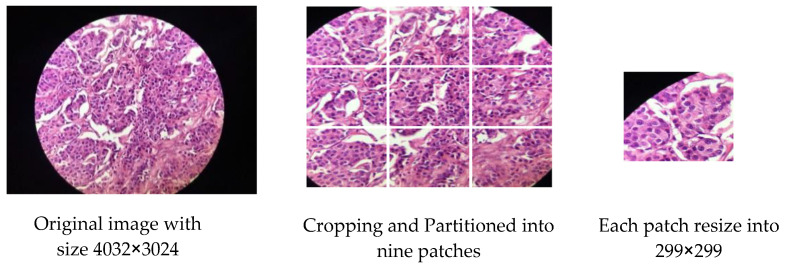
Example for Partitioning and resizing the IDC dataset.

**Figure 9:**
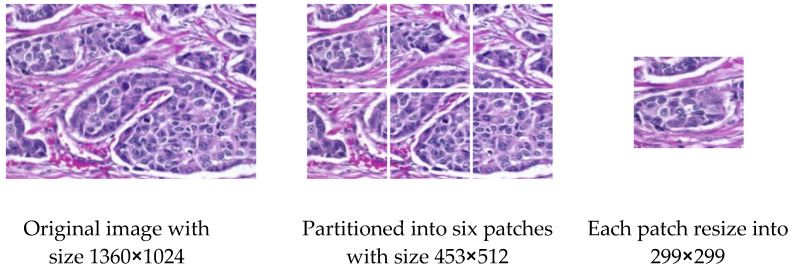
Example for Partitioning and resizing the BreCaHAD dataset.

For skin cancer, all images of ISIC2020 in the source dataset are resized to 500 × 375 to reduce image size and facilitate training. For breast cancer, some modifications are made to the source dataset. The first dataset, BreakHis, contains 7909 images, and each image is partitioned into two patches of the size of 350 × 460 (see Figure 7), so that the total number is 15818 images. The second dataset, IDC, contains 922 images; one image is cropped and partitioned into nine patches to become 8262 images after removing the images that contain a black background only (see Figure 8). The third dataset, SPIE-AAPM-NCI-BreastPathQ, contains 3698 images, while the fourth dataset, BreCaHAD, contains 162 images; each image is partitioned into six patches to become 972 images (see Figure 9). The process of partitioning the images into patches was performed due to the large image size, which may cause image distortion when resized to a smaller size. Partitioning was performed only for the unclassified images because the purpose of the unclassified images is to extract features relevant to the target disease. All datasets, from the first to fourth dataset, are resized to 299 × 299 to match with input size for all models, and all data format is changed to jpg.

In addition, some modifications are applied to the target dataset for skin cancer. The ISIC2020 dataset contains 32,542 images of the benign class and 584 Image of the malignant class; 9000 images of the benign class are taken for training for the purpose of checking how the model performs with a limited dataset. As for breast cancer, the target dataset contains the ICIAR 2018 (BACH) dataset, which contains 400 images, 100 images for each class; the images are resized to 299 × 299.

#### 2.2.2. Data Augmentation

**Figure 10:**
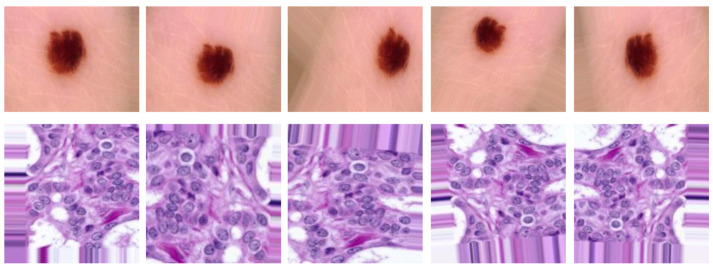
Examples of the applied data augmentation techniques.

The proposed system employed several data augmentation techniques, such as rotation, shifting, brightness, shearing, zooming, and flipping, as shown below, to overcome the problem of unbalanced data and to increase the number of samples in the dataset. Data augmentation includes a set of techniques that improve the attributes and size of datasets (see Figure 10). Thus, DL models can perform better when using these technologies. Table 2 shows some of the data augmentation parameters that are used in the proposed work.

For skin cancer, the data augmentation techniques are applied to the target dataset, and to the malignant class, only to increase the number of samples from 584 to 8988 for the purpose of balancing the source datasets. As for breast cancer, the technique of data augmentation is applied to datasets A and B to increase the number of samples in the dataset. Table 3 shows the application of the data augmentation technique to datasets.

#### 2.2.3. Data Splitting

After performing the process of preparing the data and the data augmentation technique, the datasets are separated into two sets: the training set for the purpose of training the model, and the test set for the purpose of testing the efficiency of the model’s performance on the classification, as shown in Table 4. Regarding skin cancer datasets, the source dataset (with 71,300 images) is split into 70% (56,594) images for the training set and 30% (14,706 images) for the testing set. The target dataset, which has 17,988 images, is divided using the same ratio that is used for splitting the source dataset, so the training set and the testing set will have 12,591 images and 5397 images, respectively.

The same splitting procedure that it is applied to split skin cancer datasets is followed for splitting breast cancer datasets; the source dataset, containing 93,010 images, is divided into 65,104 images for the training set and 27,906 for the testing set. As for the target dataset, which contains 20,365 images, it is divided into 14,254 images for the training set and 6111 images for testing set.

### 2.3. Select CNN Models Trained on ImageNet

In this proposed work, different models in terms of complexity and number of layers are tried, such as VGG 16, Xception, ResNet50, and MobileNetV2. Furthermore, the ImageNet dataset on which these models are trained contains natural, non-medical images, and since the early layers learn generic features such as edges and shapes, and the last layers learn specific features to the target task, so freezing the early layers and unfreezing the last layers, as shown in the following sub-sections, is done for the purpose of fine-tuning the models on unlabeled medical images from the same target domain, and for fine-tuning part of the last layers on labeled images of the target dataset. This method differs from the traditional transfer learning methods in that the pre-trained models are trained on natural images (ImageNet) and not medical images. Therefore, part of the last layers trained on these images are unfrozen to retrain them on medical images of the same type of disease. Moreover, this method does not require training the layers of the unfrozen models on classified images, but rather on unclassified images of the same disease to extract relevant features from the disaggregated dataset, thus greatly reducing the need for classified images. In addition, the proposed method does not require training the models on unclassified images from scratch (as L. Al-Zubaidi et al. performed). Only the last layers, specialized in extracting the features assigned to the target task, are trained, which reduces the need for many images and speeds up the process of training models. These models will be applied for the purpose of classifying skin cancer images, identifying the model that performs best for the purpose of classifying breast cancer images, and demonstrating that the proposed approach can be applied to any medical image task for which there are insufficient labeled images and largely unlabeled images available.

#### Modification of the Models for Classification Tasks

**Figure 11:**
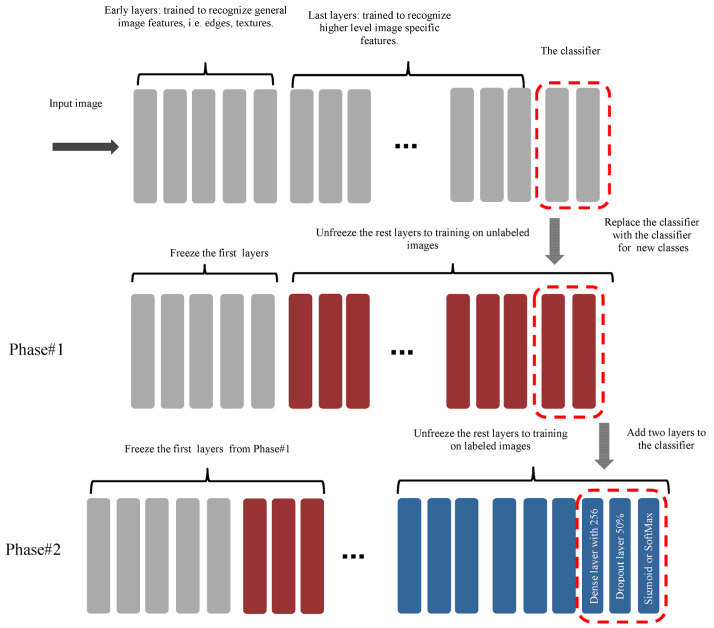
Proposed modification on the models.

All models (Vgg16, Xception, ResNet50, MobileNetV2) were modified as shown in Figure 11. 

In Phase#1: the model’s classifier for all models was replaced with a new one that fits the new task, and the early layers were frozen to save the weights; the first 11, 115, 165, and 143 layers were frozen for Vgg16, Xception, ResNet50, and MobileNetV2 models, respectively, the rest of the layers were unfrozen to train them on the source dataset.

In Phase#2: two layers were added to the classifier of each model, which is a dense layer with 256 nodes to increase classification efficiency, and a dropout layer by 50%, to reduce overfitting. After that, the first 15, 126, 171, and 149 layers of Vgg16, Xception, ResNet50, and MobileNetV2 models were frozen, respectively, and the rest of the layers were unfrozen to be trained on the target dataset.

### 2.4. Transfer Learning Process 

The transfer learning process of the proposed models for the classification of skin cancer images is carried out in two scenarios, and the same two scenarios are repeated for the classification of breast cancer images, except replacing the sigmoid activation function in the last layer with SoftMax for multiclass classification.

**Scenario1:**  Transfer learning is performed to train the classifier of the models on target dataset without fine-tuning the models on source dataset and target dataset.

**Figure 12:**
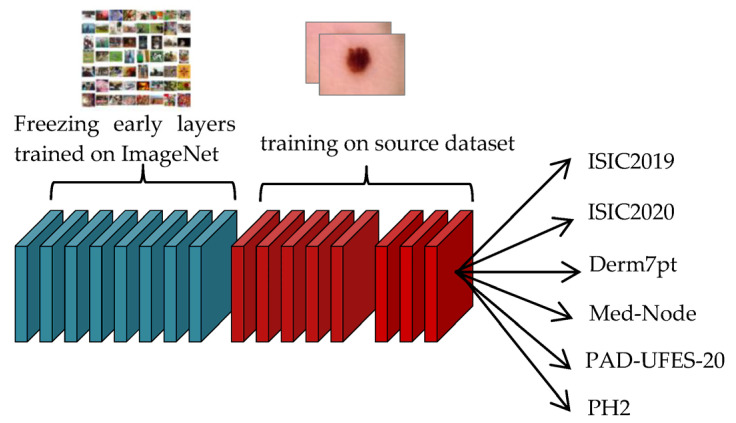
Transfer learning process of the model in the Phase#1.

**Figure 13:**
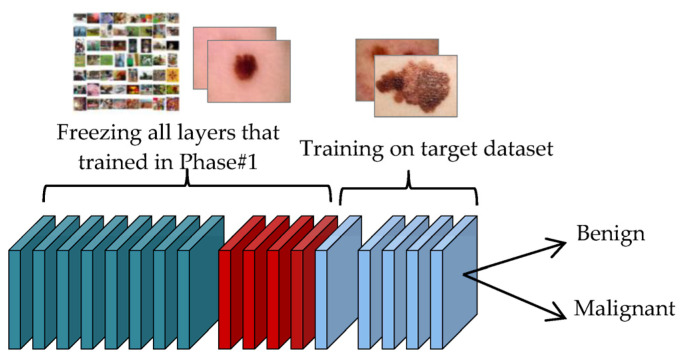
Transfer learning process of the model in the Phase#2.

**Scenario2:**  In this scenario the training process for the proposed approach to DTL is conducted in two phases:

Phase#1: At this phase, the transfer learning process of the four models is performed on unclassified images of the same disease that were collected in the source dataset, as shown in Figure 12. This step is important for the convergence of the domain between the source domain and the target domain, and to reduce the effect of ImageNet. This step is important for extracting features that are close to the target task.
Phase#2: After conducting the transfer learning process in Phase#1, the transfer learning process is performed in this phase on classified images of the target task that were collected in the target dataset, as shown in Figure 13, for the purpose of classifying skin cancer images into two classes: benign and malignant.

## 3. Results and Discussions

After performing the training of the proposed models, the testing process is performed to test the ability of these models to correctly classify the disease by testing them on the testing set within target dataset. The most common metrics were used for such cases, such as accuracy, precision, recall (sensitivity),specificity, and F1-score, to measure the performance of the models [35].

(1) $$\mathrm{Accuracy}=\frac{\mathrm{TP}+\mathrm{TN}}{\mathrm{TP}+\mathrm{TN}+\mathrm{FP}+\mathrm{FN}}$$

(2) $$\mathrm{Precision}=\frac{\mathrm{TP}}{\mathrm{TP}+\mathrm{FP}}$$

(3) $$\mathrm{Recall}=\frac{\mathrm{TP}}{\mathrm{TP}+\mathrm{FN}}$$

(4) $$\mathrm{Specificity}=\frac{\mathrm{TN}}{\mathrm{TN}+\mathrm{FP}}$$

(5) $$F1\text{-}\mathrm{score}=2\times \frac{\left(\mathrm{Precision}\;\times \mathrm{Recall}\right)}{\left(\mathrm{Precision}+\mathrm{Recall}\right)}$$

This section is divided as follows: Section 3.1 presents the experimental results of Breast Cancer Image Classification task. In Section 3.2, the experimental results of the Breast Cancer Image Classification task are presented. Finally, in Section 3.3, the obtained results are compared with other related work.

### 3.1. The Experimental Results for the Classification of Skin Cancer Images in the ISIC2020 Dataset

In this section, the transfer learning performance of the four models (VGG16, Xception, ResNet50, and MobileNetV2) using Scenario1 and Scenario2 on the testing set of the target dataset for the skin cancer classification task will be demonstrated. Moreover, performance will be compared with three different sampling cases: Firstly, performance without augmentation techniques and without balancing classes; secondly, performance without data augmentation and with balancing classes; and thirdly, performance comparison with data augmentation techniques.

Hyperparameters, shown in Table 5, are selected to train the models for both scenarios. Two layers are added to all the model’s classifier, which is a dense layer containing 256 nodes to better improve the performance of the classifier. This number of nodes is selected according to the experiments that are applied to a different number of nodes (2048, 1024, 512, 256 and 128), and the models’ performance was better with 256 nodes. In addition, a 50% dropout layer has been added to reduce overfitting, as it is found to be the best one after experimenting with the two most used ratios in the literatures (20% and 50%) in a dropout layer. The number of trainable layers and the number of frozen layers is selected according to experiments, and the values are fixed accordingly. The batch size of 64 is chosen to pass 64 images for each iteration during the training process, after trying different batch sizes (32, 64 and 128). Experiments proved that the batch size of 64 is the best one, because the batch size of 32 increases the number of iterations for each epoch, which slows down the training process, while a batch size of 128 requires more memory. The number of epochs was 30 after conducting several experiments on several different epochs. During the experiments, the selected number of epochs is proven to be good to produce good results and prevent overfitting. A learning rate of 0.0001 for Scenario1 and 0.000001 for Scenario2 are chosen because of the unfreezing of the last layers in Scenario2 for the purpose of training, which requires a lower learning rate for fine-tuning. These values are chosen based on experiments. To prevent overfitting, early stopping with patience 8 is added after trying different values (4, 5, and 8). Experiments have shown that early stopping with patience of 8 is good for preventing the occurrence of overfitting and giving an opportunity for the model to improve. The following subsections describe experimental results. 

**Figure 14:**
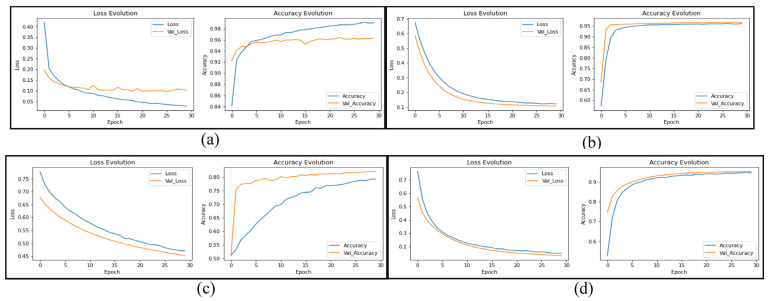
Learning curves of the models: (**a**) VGG16; (**b**) Xception; (**c**) ResNet50; (**d**) MobileNetV2 to transfer learning from Scenario2 for skin cancer classification with data augmentation.

**Figure 15:**
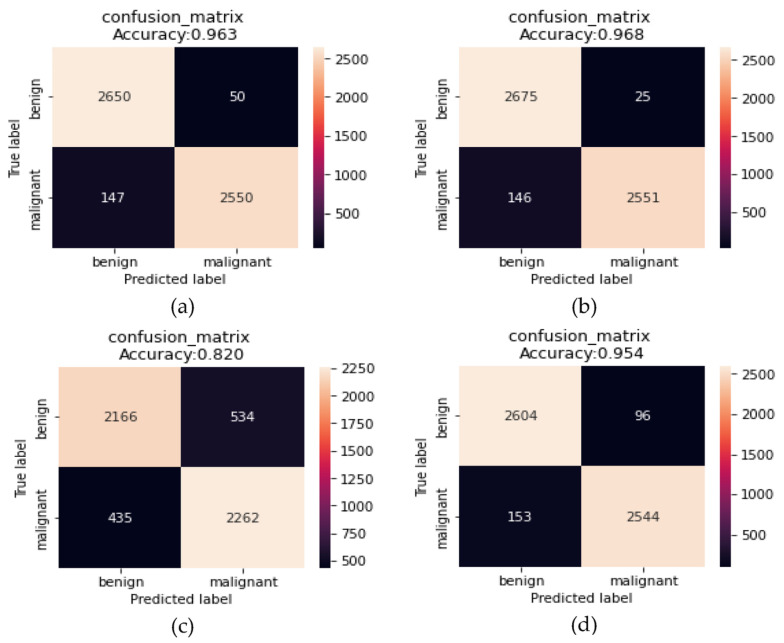
Confusion matrix of the models: (**a**) VGG16; (**b**) Xception; (**c**) ResNet50; (**d**) MobileNetV2 to transfer learning from Scenario2 for skin cancer classification with data augmentation.

**Figure 16:**
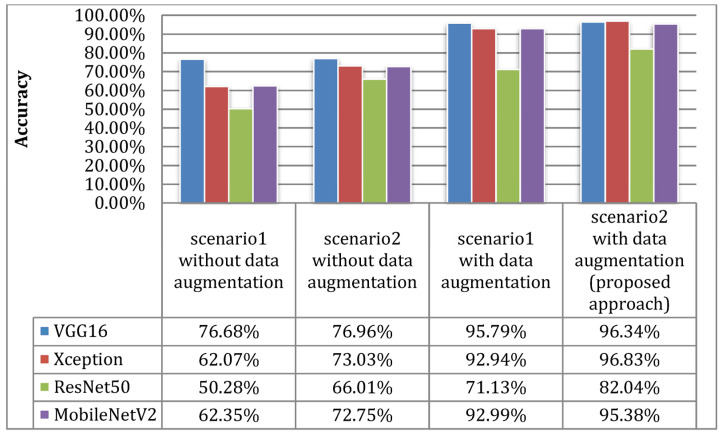
Comparing the accuracy metric of Scenario1 and Scenario2 with and without the use of data augmentation.

The results in Figure 14 and Figure 15 show the improvement of all the proposed models, which proves that the use of the proposed DTL has significantly improved the performance of the four models for classifying skin cancer images. The fine-tuning of the last layers of the model on a large number of unclassified images of the same disease, and the transfer learning procedure in the second stage on a small number of classified images as a result of the domain convergence, reduced the effect of ImageNet features. In addition, the design of the new models helped improve the performance of classifiers and provided better prediction by adding a hidden layer with 256 nodes, while solving the problem of overfitting using dropout layers by 50%, and as shown in Figure 16. Scenario2 improved the performance of the VGG16 model by 0.28%, the Xception model by 10.96%, the ResNet50 model by 15.73%, and the MobileNetV2 model by 10.4% without data augmentation. It improved the VCG16 model by 19.66%, the Xception model by 34.76%, the ResNet50 model by 31.76%, and the MobileNetV2 model by 33.03% with data augmentation.

The slight improvement in the VGG16 model is attributed to the high number of parameters of the model, which require a large number of images to perform better.

**Figure 17:**
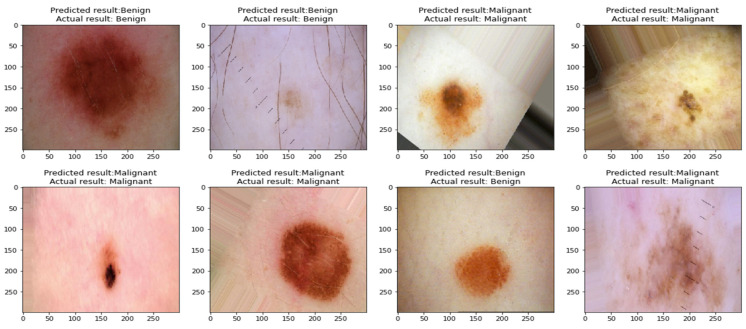
Images classified correctly by the proposed classifier.

The obtained results show that the Xception model performed the best compared to the remaining models when classifying skin cancer images in the ISIC2020 dataset, as it obtained an accuracy of 96.83%, a precision of 96.919%, a recall of 96.826%, an F1-score of 96.825%, a sensitivity of 99.07%, and a specificity of 94.58%. To prove that the results of this model did not come from the effect of random weights, the process of implementing the test was repeated for five times, as shown in Table 6, which shows the convergence of the results, which indicates the stability of the model in the prediction process. Figure 17 shows benign and malignant images correctly predicted by the classifier of the proposed approach.

### 3.2. The Experimental Results for the Classification of Breast Cancer Images in the ICIAR2018 Dataset

**Figure 18:**
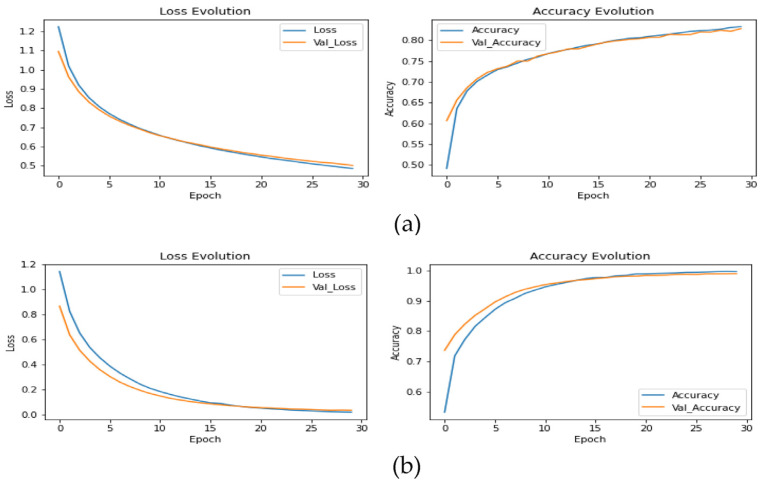
Learning curves of the scenarios: (**a**) Scenario1; (**b**) Scenario2 for breast cancer classification with data augmentation.

After training the model as shown in Figure 18 on the training set that contains 14,254 images (70% of 20,365 images) within the ICIAR2018 source dataset after data augmentation, the obtained results are: Accuracy of 82.48%, precision of 82.798%, recall of 82.840%, F1-score of 82.764%, sensitivity of 74.04%, and specificity of 85.77% using scenario1, and accuracy of 99%, precision of 99.003%, recall of 98.995%, F1-score of 99%, sensitivity of 98.55%, and specificity of 99.14% using Scenario2 when tested on 6111 images (30% of 20,365 images) within the testing set as shown in Table 7.

**Figure 19:**
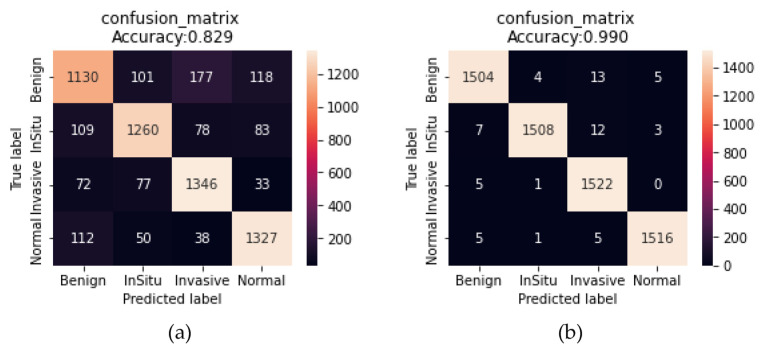
Confusion matrix of the scenarios: (**a**) Scenario1; (**b**) Scenario2 for breast cancer classification with data augmentation.

These results demonstrate the success of the proposed approach to transfer learning, with regard to model fine-tuning on unclassified images of the same disease, with the fine-tuning of a number of final layers on classified images within the target dataset, which helps with the convergence of the features of the source domain with the target domain compared to the features extracted from ImageNet, as shown in Figure 19. In addition, the entire process of preserving the image without partitioning it into patches helped in saving some important information that may be lost when partitioning the images into small patches, by working to increase the images only by using data augmentation techniques that have proven their efficiency to solve the problem of lack of images and avoid overfitting.

### 3.3. Compare Our Results with Other Related Work

This section compares and discusses the results obtained using the proposed approach with the most recent related work.

#### 3.3.1. Comparison of Skin Cancer Image Classification Results with Other Works

The comparison and discussion the of results of the proposed approach with the related works to classify skin cancer images on the ISIC2020 dataset has been presented. Table 8 shows that the proposed approach ranked second after L. Alzubaidi et al. (2021) [10], where they obtained an accuracy of 98.57%, a precision of 99.18%, a recall of 97.90%, and an F1 score of 98.53%. Z. M. Arkah (2021) [18] ranked third with an accuracy of 93.7%, a precision of 95.7%, a recall of 94.6%, and an F1-score of 95.1%. V. Shah et al. (2020) [15] came in fourth, with an accuracy of 93.96%, a precision of 94.11%, a recall of 93.96%, an F1-score of 93.24%, a sensitivity of 99.7%, and a specificity of 55.67%. R. Kaur et al. (2022) [19] came in fifth place, with an accuracy of 90.42%, a precision of 90.48%, a recall of 90.39%, an F1-score of 90.41%, and a specificity of 90.39%. R. Zhang (2021) [17] and C. Li et al. (2021) [16] ranked sixth and seventh, with AUC-ROC 0.917 and 0.909, respectively.

According to V. Shah et al. (2020), C. Li et al. (2021) and R. Zhang (2021) used traditional transfer learning methods based on the use of pre-trained models on ImageNet as feature extractors. These old methods suffer from field mismatch between the target field images and features extracted from ImageNet images, and in addition to that, their models suffer from an overfitting problem. Thus, Z. M. Arkah (2021) and L. Alzubaidi et al. (2021) conducted a different study to solve the domain mismatch problem, whereas M. Arkah (2021) used the ResNet50 model and trained it from scratch on unclassified images and then carried out transfer learning on classified images of the target task to get rid of the effect of the features extracted from the ImageNet and to take advantage of the extracted features of unclassified images of the same disease. L. Alzubaidi et al. (2021) performed the same method used by M.Arkah, but by building a deep convolutional neural network inspired by different models. It is expected that these methods will lead to higher performance due to the large convergence of the field between the source field and the target field, but it needs a huge amount of unclassified images to train the models from scratch; they trained the model on (200,000 unclassified images). In addition to that, it takes a long training time and requires high computational power to train the models. Therefore, the proposed method for transfer learning based on a DTL procedure for the modified models is an excellent solution that does not require such a huge amount of unclassified images. In addition, it does not require a long training time, by performing fine-tuning of the models on unclassified images (71,294 images) for the last layers only after unfreezing them, instead of training from scratch, taking advantage of the features extracted from the early layers trained on ImageNet as generic feature extractors, and thus we have achieved excellent results with the least number of unclassified images. In addition, modifying the models with the new design significantly improved the performance of classifiers predictors and solved the problem of overfitting. Moreover, previous studies were not comprehensive of all performance measures, as our work is comprehensive of all important measures used to measure the performance of models. It is worth mentioning that all the comparisons are conducted using the same preprocessing method with different transfer learning methods.

#### 3.3.2. Comparison of Breast Cancer Image Classification Results with Other Works

The results in Table 9 demonstrate the superiority of the proposed approach to classify breast cancer images for the ICIAR2018 dataset, as our proposal ranked first with an accuracy of 99%. L. Alzubaidi et al. (2021) [10] came second with an accuracy of 97.51%. T. Kausar et al. (2019) [21] ranked third with an accuracy of 94.3%. S. H. Kassani et al. (2019) [20] ranked fourth with an accuracy of 92.5%. C. P. Nguyen et al. (2019) [22] ranked fifth with an accuracy of 78%. All the comparisons are conducted using the same preprocessing method with different transfer learning methods.

The results show the strong performance of the proposed approach at all metrics, compared to all of the researchers who relied on the accuracy metric only in evaluating their models; this does not give a comprehensive insight of the nature of the predictors of the classifier between the classes. The obtained results indicate the success of the proposed work in preserving the whole image without partitioning it into small patches, in order to preserve the important information that could be lost when partitioning it into patches. In addition, to solve the problem of the small number of images, we used data augmentation techniques, which generate new images with different angles of rotation, flipping, and zooming from the original images, which can occur from tissue imaging by specialists.

**Figure 20:**
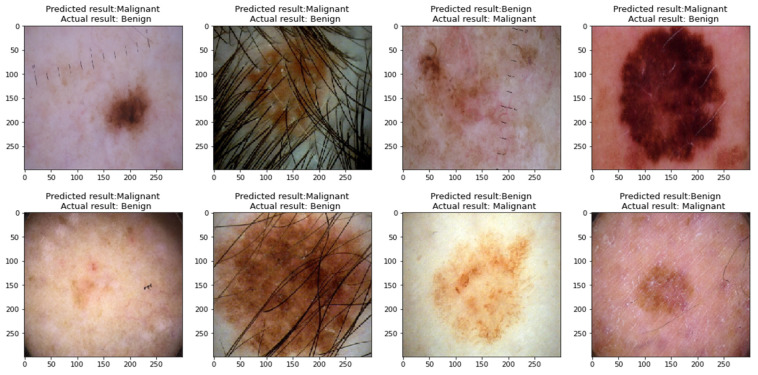
Misclassified images by the classifier of the proposed approach.

In most studies, there are some limitations that can be solved in future research. This research includes some limitations because of the scope of the study. The limitations of this research are:

1- The proposed models for classifying skin cancer images still incorrectly predicted some images; this is because some skin cancer images contain thick hair covering the affected area, in addition to the presence of some color labels next to the affected area and the presence of some light reflections on the surface of the skin, as shown in Figure 20, which hinders the process of interpretation image.
2- Even when using the proposed approach, there is still a problem of biasing classifiers to the category with the largest number of samples when using an unbalanced dataset.

## 4. Conclusions and Future Work

This study presented a proposed approach to solve the problem of the lack of labeled medical images by including transfer learning methods on pre-trained models on ImageNet (VGG16, Xception, ResNet50, MobileNetV2). To obtain the extracted features that are closer to the target task, the last layers of the models are unfrozen and trained on a set of unlabeled images for the same type of disease, and part of the last layers on labeled images, to better improve performance, in addition to the use of data augmentation techniques to increase the number of images and for balancing the classes of the dataset. The proposed approach is applied to classify the images of the ISIC2020 skin cancer dataset into two classes, benign and malignant, and to classify the images of the ICIAR 2018 breast cancer dataset into four classes: invasive carcinoma, in situ carcinoma, benign tumor, and normal tissue.

The obtained results showed an improvement in the performance of the models after fine-tuning them on a large set of unlabeled images and on a small set of labeled images for skin cancer image classification tasks, where the performance of the VGG16 model improved by 0.28%, the Xception model by 10.96%, the ResNet50 model by 15.73%, and the MobileNetV2 model by 10.4% without data augmentation, while improving the VCG16 model by 19.66%, the Xception model by 34.76%, the ResNet50 model by 31.76%, and the MobileNetV2 model by 33.03% with data augmentation. The Xception model obtained the highest performance compared to the rest of the models when classifying skin cancer images in the ISIC2020 dataset, as it obtained accuracy of 96.83%, precision of 96.919%, recall of 96.826%, F1-score of 96.825%, sensitivity of 99.07%, and specificity of 94.58%. To prove that the proposed approach is applicable to more than one type of medical image, the approach is applied to classify the images of the ICIAR 2018 dataset for breast cancer. The Xception model obtained accuracy of 99%, precision of 99.003%, recall of 98.995%, F1-score of 99%, sensitivity of 98.55%, and specificity of 99.14%. We compared this with the use of traditional transfer learning methods with data augmentation technology, which obtained accuracy of 82.48%, precision of 82.798%, recall of 82.840%, F1-score of 82.764%, sensitivity of 74.04%, and specificity of 85.77%, which proves the success of the proposed approach in all our experiments. 

The suggestions for future work: For better performance, a larger number of the last layers can be unfrozen and trained on a larger number of unlabeled images. Executing fine-tuning on the models by training the last layers on images like the target images, for example, microscopic images of colon and bone cancer, can be used to improve the performance of the tasks of classifying breast cancer images due to the similarity of the images in the histological structure, which can be used to extract features that are like the features of breast cancer. Some improvements could be made to skin cancer images, such as removing hair from the image, cropping the background, and keeping the area of interest.

## Figures and Tables

**Table 1:** Techniques used in the skin cancer and the breast cancer classification tasks.

Author(s)/Year	Methods/Techniques	Cancer Type	Dataset	Strengths	Weaknesses
(V. Shah et al., 2020) [15]	“Transfer learning on (DenseNet-121, SE-ResNeXt50, ResNet50, and VGG19)”	Skin cancer	ISIC2020	“ResNet-50 according to sensitivity, specificity, and obtained the best accuracy results among the other three, with values of 99.7%, 55.67%, and 93.96%, respectively.”	It is pointless to use a test with low specificity for diagnosis because many people without the disease will show positive results and potentially receive unnecessary diagnostic procedures.
(C. Li et al., 2021) [16]	Transfer learning on three models (EfficientNet-B4, vgg16, and ResNet50)	Skin cancer	ISIC2020	“They had an AUC-ROC score for EfficientNet-B4 of 0.909, which is 3.5% higher than VGG16 and 2.3% higher than Resnet50.”	They did not experiment with the effect of balancing the classes, because the ISIC2020 dataset suffers from the problem of imbalance between the benign and malignant classes. In addition, the proposed model suffers from the problem of overfitting.
(R. Zhang 2021) [17]	Transfer learning on EfficientNet-B6	Skin cancer	ISIC2020	AUC-ROC score of 0.917.	The model suffers from an overfitting problem, so the technique of data augmentation and adding dropout layers can be used to solve this problem.
(Z. M. Arkah 2021) [18]	Transfer learning on (VGG, GoogleNet, ResNet50)	Skin cancer	ISIC2020	“The ResNet 50 model had accuracy, precision, recall, and F1 scores of 93.7%, 95.7%, 94.6%, and 95.1%, respectively.”	The process of training from scratch takes time and requires a very large number of images, so the process of fine-tuning the pre-trained models for some of the last layers that extract customized features may lead to better results and less training time.
(L. Alzubaidi et al., 2021) [10]	Transfer learning on deep convolutional neural network (DCNN)	Skin cancer	ISIC2020	The proposed model achieved an F1 value of 89.09% when training from scratch and 98.53% with the proposed method	The process of training from scratch performs better but takes a lot of time to train and requires a lot of images to train well.
(R. Kaur et al., 2022) [19]	Transfer learning on deep convolutional neural network (DCNN)	Skin cancer	ISIC2020-ISIC2016-ISIC2017	“The proposed DCNN achieved average ACC, PRE, and REC of 81.41%, 81.88%, and 81.30% on ISIC 2016, of 88.23%, 78.55%, and 87.86% on ISIC 2017, and of 90.48%, 90.39%, and 90.42% on ISIC 2020.”	“The designed DCNN model can be further extended to multi-class classification to predict other different types of skin cancers.”
(S. H. Kassani et al., 2019) [20]	Transfer learning on Xception	Breast cancer	ICIAR 2018	Their proposed model using a pre-trained Xception model obtained 92.50% average classification accuracy.	The accuracy measure alone is not sufficient to evaluate the model, so other measures such as precision, recall, and F1 score can be used.
(T. Kausar et al., 2019) [21]	Transfer learning on vgg16	Breast cancer	ICIAR 2018	They achieved an accuracy of 94.3% for the multi-class classification.	The effect of dataset size with or without data augmentation on classification is not reported
(C. P. Nguyen et al., 2019) [22]	CNN is taken from the design principle of DenseNet	Breast cancer	ICIAR 2018	78% accuracy	Data augmentation techniques using GAN to generate additional datasets have not been considered.”
(L. Alzubaidi et al., 2021) [10]	Transfer learning on deep convolutional neural network (DCNN)	Breast cancer	ICIAR 2018	Achieved an accuracy value of 97.51%	In patch extraction, some cells are split between adjacent patches, and these cut cells cause incorrect classifications in the detection results. There is also no guarantee that small patches will contain enough information for the correct class.

**Table 2:** Data augmentation parameters.

Augmentation Parameter	Value	Description
Rotation range	10	Random rotation between 0 and 10.
Width shift range	0.2	Randomly shifts images in the horizontal direction by 0.2
Height shift range	0.2	Randomly shifts images in vertical direction by 0.2
Brightness range	[0.1, 1.5]	Randomly changes the brightness of the image.
Shear range	0.2	Shear the image by 20%.
Zoom range	1.2	Zoom in 20% from the center.
Vertical flip	True	Randomly flip the image in vertical direction.
Horizontal flip	True	Randomly flip the image in horizontal direction.
Fill mode	nearest	Fills the empty values by the closest pixel value.

**Table 3:** Data augmentation to increase the samples of the datasets after the data preparation step.

Dataset	Class	Before Data Augmentation	After Data Augmentation
ISIC2020/target dataset	Benign	9000	Not applied
	Malignant	584	8988
ISIC2019/source dataset	Unlabeled	33569	Not applied
ISIC2020/source dataset	Unlabeled	33126	Not applied
Derm7pt/source dataset	Unlabeled	1937	Not applied
PH2/source dataset	Unlabeled	200	Not applied
PAD-UFES-20/source dataset	Unlabeled	2298	Not applied
MED-NODE/source dataset	Unlabeled	170	Not applied
BreakHis/source dataset	Unlabeled	15818	31635
IDC/source dataset	Unlabeled	8262	30581
SPIE-AAPM-NCI BreastPathQ/source dataset	Unlabeled	3698	22187
BreCaHAD/source dataset	Unlabeled	972	8607
ICIAR 2018/target dataset	Invasive carcinoma	100	5093
	In situ carcinoma	100	5098
	Benign	100	5085
	Normal	100	5089

**Table 4:** Splitting the datasets.

Datasets	Dataset Type	Total Samples 100%	Training Set 70%	Testing Set 30%
Source dataset	Skin cancer	71,300	56,594	14,706
Target dataset	Skin cancer	17,988	12,591	5397
Source dataset	Breast cancer	93,010	65,104	27,906
Target dataset	Breast cancer	20,365	14,254	6111

**Table 5:** Hyperparameters selection for Scenario1 and Scenario2.

Models	Parameters	Scenario1	Scenario2	
			Phase#1	Phase#2
VGG16	Total layers	21	21	23
	Trainable layers	4	10	6
	Batch Size	64	64	64
	Epochs	30	40	30
	Learning Rate	0.0001	0.000001	0.000001
	Early Stopping	8 patience	8 patience	8 patience
	Optimizer	Adam	Adam	Adam
Xception	Total layers	134	134	136
	Trainable layers	2	19	10
	Batch Size	64	64	64
	Epochs	30	40	30
	Learning Rate	0.0001	0.000001	0.000001
	Early Stopping	8 patience	8 patience	8 patience
	Optimizer	Adam	Adam	Adam
ResNet50	Total layers	177	177	179
	Trainable layers	2	12	8
	Batch Size	64	64	64
	Epochs	30	40	30
	Learning Rate	0.0001	0.000001	0.000001
	Early Stopping	8 patience	8 patience	8 patience
	Optimizer	Adam	Adam	Adam
MobileNetV2	Total layers	156	156	158
	Trainable layers	2	13	9
	Batch Size	64	64	64
	Epochs	30	40	30
	Learning Rate	0.0001	0.000001	0.000001
	Early Stopping	8 patience	8 patience	8 patience
	Optimizer	Adam	Adam	Adam

**Table 6:** Repeated tests to measure the performance of the Xception model for classification of skin cancer images.

Execution No.	Precision (%)	Recall (%)	F1-score (%)	Accuracy (%)	Sensitivity (%)	Specificity (%)
Execution1	96.759	96.676	96.680	96.68	98.77	94.58
Execution2	96.709	96.621	96.625	96.62	98.77	94.47
Execution3	96.759	96.676	96.680	96.68	98.77	94.58
Execution4	96.784	96.696	96.695	96.7	98.88	94.51
Execution5	96.919	96.826	96.825	96.83	99.07	94.58
min_scale	96.709	96.621	96.625	96.62	98.77	94.47
max_scale	96.919	96.826	96.825	96.83	99.07	94.58

**Table 7:** Results of Scenario1 and Scenario2 for the breast cancer classification task on dataset ICIAR2018.

Method	Class	Precision (%)	Recall (%)	F1-Score (%)	Support	Accuracy (%)	Sensitivity (%)	Specificity (%)
Scenario1 with Xception	invasive	79.4	74.04	76.63	5093	82.48	74.04	85.77
	in situ	84.67	82.35	83.49	5098			
	benign	82.12	88.08	85	5085			
	normal	85	86.9	85.94	5089			
	macro-avg	82.7975	82.8425	82.765	20365			
	weighted-avg	82.798	82.840	82.764	20365			
Scenario2 with Xception	invasive	98.88	98.55	98.72	5093	99	98.55	99.14
	in situ	99.6	98.56	99.08	5098			
	benign	98.06	99.6	98.83	5085			
	normal	99.47	99.27	99.37	5089			
	macro-avg	99.003	98.995	99	20365			
	weighted-avg	99.003	98.995	99.000	20365			

**Table 8:** Comparison of the results of the proposed approach with related works for the classification of skin cancer images in the ISIC2020 dataset.

Author	Method	Precision (%)	Recall (%)	F1-Score (%)	Accuracy (%)	Sensitivity (%)	Specificity (%)	AUC-ROC
V. Shah et al. (2020) [15]	Transfer learning on ResNet50 using fine-tuning	94.11	93.96	93.24	93.96	**99.7**	55.67	N/V
C. Li et al. (2021) [16]	Transfer learning on EfficientNet-B4	N/V	N/V	N/V	N/V	N/V	N/V	0.909
R. Zhang (2021) [17]	Transfer learning on EfficientNet-B6	N/V	N/V	N/V	N/V	N/V	N/V	0.917
Z. M. Arkah (2021) [18]	Transfer learning on ResNet50 (Training the model from scratch on unclassified images then performing transfer learning on classified images)	95.7	94.6	95.1	93.7	N/V	N/V	N/V
L. Alzubaidi et al. (2021) [10]	Transfer learning on DCNN (Training the model from scratch on unclassified images then performing transfer learning on classified images)	99.18	97.90	98.53	98.57	N/V	N/V	N/V
R. Kaur et al. (2022) [19]	Transfer learning on DCNN	90.48	90.39	90.41	90.42	N/V	90.39	N/V
Proposed approach with Xception	Transfer learning on Xception (Training the last layers on unclassified images then training the classifier on classified images)	96.919	96.826	96.825	96.83	99.07	94.58	0.9896

**Table 9:** Comparison of the results of the proposed approach with related works for the classification of breast cancer images in the ICIAR-2018 dataset.

Author	Method	Precision (%)	Recall (%)	F1-Score (%)	Accuracy (%)	Sensitivity (%)	Specificity (%)
S. H. Kassani et al. (2019) [20]	Transfer learning on Xception (They combined the features from the layers (26, 36 and 126) and then applied a GAP to the extracted features, and then combined them together to produce the final feature vector)	N/V	N/V	N/V	92.5	N/V	N/V
T. Kausar et al. (2019) [21]	Transfer learning on vgg16 (The FC layers are removed in the original model so that this model can accept images of random size They applied process (GAP) over the output features of convolution layers C3_3, C4_3, and C5_3.)	N/V	N/V	N/V	94.3	N/V	N/V
C. P. Nguyen et al. (2019) [22]	CNN is taken from the design principle of DenseNet	N/V	N/V	N/V	78	N/V	N/V
L. Alzubaidi et al. (2021) [10]	Transfer learning on DCNN (Training the model from scratch on unclassified images then performing transfer learning on classified images)	N/V	N/V	N/V	97.51	N/V	N/V
Proposed approach with Xception	Transfer learning on Xception (Training the last layers on unclassified images then training the classifier on classified images)	99.003	98.995	99	99	98.55	99.14

## Data Availability

The data supporting reported results can be found and publicly archived datasets analyzed from [23,24,25,26,27,28,29,30,31,32,33,34].
